# Massively parallel functional profiling prioritizes breast cancer risk variants and implicates *CCDC88C* in ER-positive breast cancer

**DOI:** 10.1016/j.xhgg.2026.100674

**Published:** 2026-09-17

**Authors:** Kate Mackie, Harriet Kemp, Andrea Gunnell, James B. Studd, Molly Went, Philip Law, Katarzyna Tomczyk, Rafaela Ferreira, Ernesto Lopez, Selin Sevgi, Yunjiao Lu, Alisa Zvereva, Nick Orr, Richard S. Houlston, Nichola Johnson, Olivia Fletcher, Syed Haider

**Affiliations:** 1 The Breast Cancer Now Toby Robins Research Centre, The Institute of Cancer Research, London SW3 6JB, UK; 2 Division of Genetics and Epidemiology, The Institute of Cancer Research, London SW7 3RP, UK; 3 The Patrick G Johnston Centre for Cancer Research, Queen’s University Belfast, Belfast BT9 7AE, UK

**Keywords:** breast cancer, risk targets, MPRA profiling

## Abstract

Genome-wide association studies (GWASs), combined with fine-mapping, have identified 196 independent signals associated with breast cancer risk. Deciphering the functional basis of these associations can inform our understanding of the biology and etiology of breast cancer. Decoding GWAS risk associations is challenging due to linkage disequilibrium between variants and because most variants map to non-coding regions, influencing breast cancer risk via *cis*-regulatory mechanisms that modulate the expression of target genes. To prioritize variants with allele-specific regulatory activity at breast cancer risk loci, we carried out a lentivirus-based massively parallel reporter assay (lentiMPRA) to screen 5,116 credible causal variants (CCVs) across these signals. We identified 709 variants mapping to 140 risk regions that are associated with significant variation between reference (REF) and alternative (ALT) alleles. A follow-up investigation at 14q32.11 revealed that rs7153397 may influence breast cancer risk through expression of *CCDC88C*, which in turn could impact prognosis. These findings provide a prioritized set of functional variants for downstream analyses, advancing our understanding of breast cancer risk mechanisms.

## Teaser

Translating GWAS findings requires identifying functional variants and target genes. Using lentiMPRA, we screened 5,116 variants across 195 breast cancer risk signals, finding 709 with allele-specific enhancer activity. We showed that rs7153397 impacts *CCDC88C* expression, potentially influencing risk and prognosis. This work provides a basis for prioritizing variants in future studies.

## Introduction

Breast cancer, which affects over 2 million women worldwide annually, has a strong heritable basis. Genome-wide association studies (GWASs) of breast cancer, combined with fine-mapping, have identified 196 independent signals associated with breast cancer risk.^1^ Deciphering the functional basis of these risk associations has the potential to inform on the biology and etiology of breast cancer. However, decoding GWAS risk associations is challenging due to linkage disequilibrium (LD) between variants and because most variants localize to non-coding regions.

Fine-mapping analysis by the Breast Cancer Association Consortium (BCAC) and the Consortium of Investigators of Modifiers of BRCA1/2 (CIMBA) reported 7,394 credible causal variants (CCVs) as the functional basis of these 196 associations.^1^ CCVs are defined as variants with an association *p* value within two orders of magnitude of an index variant.^2^ While such computational approaches can prioritize variants and predict candidate target genes using *in silico* data, experimental validation is required to confirm regulatory activity and target genes. Most non-coding GWAS risk variants are thought to act via *cis*-regulatory mechanisms that modulate the expression of their target genes. By examining transcriptional changes linked to genetic variants, specific alleles can be associated with altered gene expression.

While classical reporter assays are limited to assessing the allelic activity of individual variants, massively parallel reporter assays (MPRAs) offer a scalable approach to evaluate the regulatory effects of thousands of variants simultaneously. To systematically assay the allele-specific regulatory activity associated with variants at breast cancer risk loci, we carried out a lentivirus-based MPRA (lentiMPRA)^3^ to screen 5,116 of the BCAC-reported CCVs across 195 of the independent risk signals (1–351 CCVs per signal).^1^

Here, we report the results of this lentiMPRA analysis in T-47D breast cancer cells. We identify a total of 709 CCVs across 140 risk regions that exhibit significant allelic differences in regulatory activity in T-47D cells. Follow-up analysis of rs7153397 at 14q32.11 identifies *CCDC88C* as the target gene at this locus, with its expression associated with breast cancer subtype and survival in estrogen receptor-positive (ER+) cases.

## Results

To determine which CCVs reported by BCAC^1^ are functional, we adapted a lentiMPRA to screen 5,116 CCVs across 195 independent risk signals in the breast cancer cell line T-47D. We generated a plasmid library of 20,878 oligonucleotides, each 270 bp centered on the reference (REF) or alternative (ALT) alleles in forward and reverse orientations, linked to unique barcodes (Figure 1A). The library was packaged into lentivirus and used to infect T-47D breast cancer cells in triplicate, then sequenced to assess regulatory activity via barcode transcription^3^ (Figure 1A). T-47D cells were chosen for their ER+ profile and availability of epigenetic (DNase I hypersensitive sites [DHSs], H3K27ac, and transcription factor [TF] chromatin immunoprecipitation sequencing [ChIP-seq]) data.

**Figure 1:**
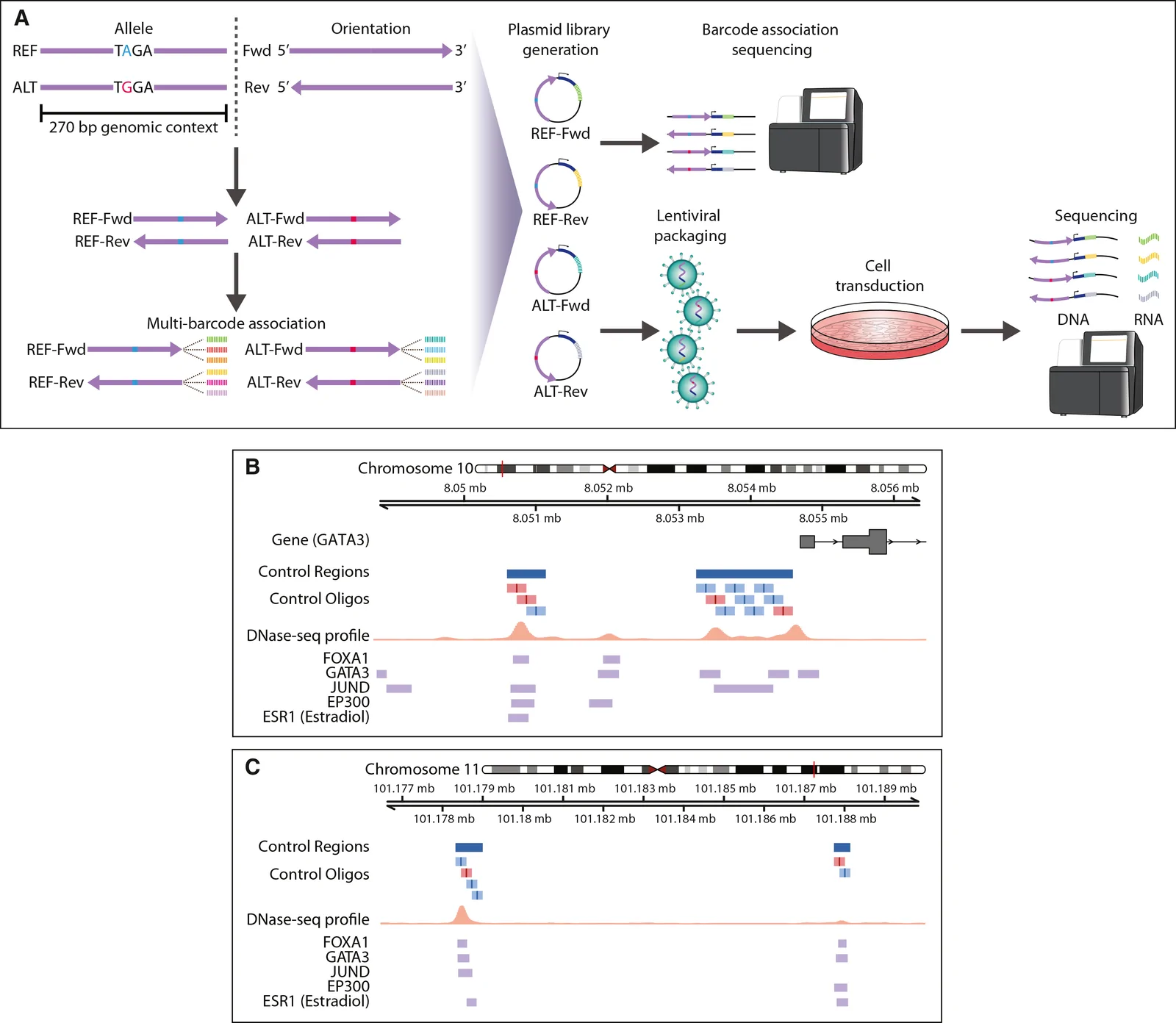
LentiMPRA study design (A) Oligonucleotides containing reference (REF) or alternative (ALT) allele and in forward (Fwd) or reverse (Rev) orientation were linked to unique barcodes to generate an oligonucleotide library. Barcoded oligonucleotides were inserted into plasmids and packaged into lentivirus. T-47D cells were infected, and DNA and RNA sequencing were used to assess regulatory activity. (B and C) Genomic regions near *GATA3* (B) and *PGR* (C) with oligonucleotides colored according to enhancer activity (red: significant, blue: non-significant) and T-47D annotations. DNase sequencing profile, orange; TF ChIP-seq, purple.

As positive controls, we designed oligonucleotides targeting regions near *GATA3* and *PGR* (Figures 1B and 1C), known for their regulatory roles in ER+ breast cancer.^4^^,^^5^ These comprised 36 oligonucleotides across regions with DHS and TF binding (*FOXA1*, *GATA3*, *JUND*, *EP300*, and *ESR1*; Figures 1B and 1C). Negative controls comprised 400 oligonucleotides, from 100 scrambled sequences. The library contained 3.64 million unique barcodes, covering 99.16% of designed oligonucleotides (mean: 175 barcodes per oligonucleotide). Three replicates showed strong correlation (r > 0.97; Figure S1).

We used MPRAnalyze^6^ to quantify enhancer activity (alpha) and differential activity between alleles. After filtering oligonucleotides with <20 or >200 barcodes, we analyzed 4,732 (92.49%) test CCVs, 17 (94.44%) positive controls, and 99 (99%) negative controls. Among controls, 35.29% of positive controls and 15.5% of negative controls showed significant enhancer activity (alpha, false discovery rate [FDR]-adjusted *p* < 0.1). For test CCVs, 6.47% (306/4,732) showed significant enhancer activity, implying these CCVs mapped to regulatory sequences (Figure 2A).

**Figure 2:**
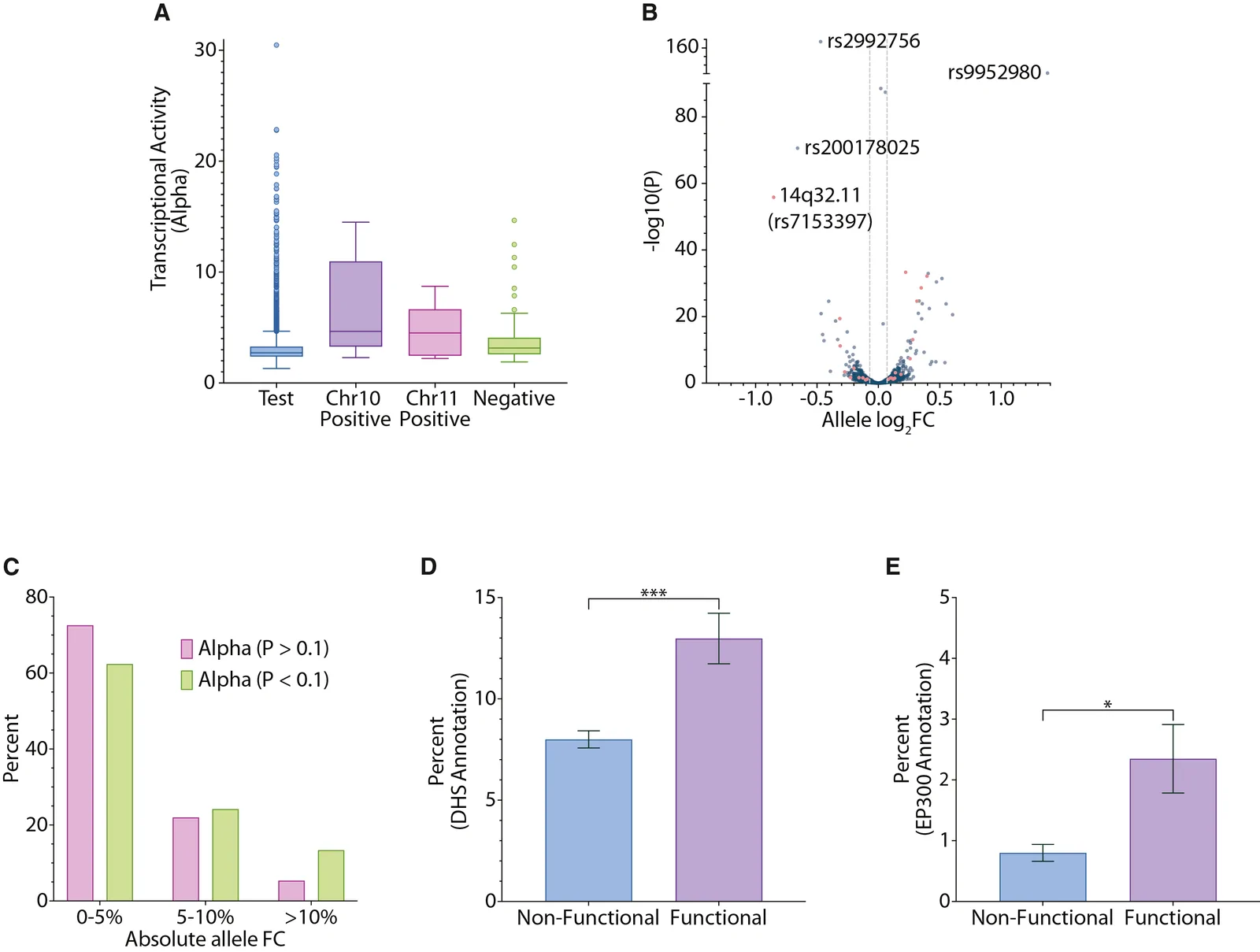
LentiMPRA identifies functional breast cancer-associated risk variants (A) Boxplot of enhancer activity for test, positive, and negative control sequences. The box represents the interquartile range (IQR), with whiskers 1.5× the IQR; the center line represents the median. (B) Volcano plot of allele log_2_FC vs. −log10 of FDR-corrected *p* for test sequences, with vertical lines at ±0.07 log_2_FC (equivalent to a ±5% difference in activity between alleles). Red dots indicate 23 variants prioritized for follow-up. (C) Proportion of variants with increasing absolute allele FC, stratified by enhancer activity *p* = 1.51 × 10^−8^. (D and E) Percentage of sequences with DHS (D) or *EP300* (E) annotations comparing non-functional (allele log_2_FC *p* > 0.1) and functional (allele log_2_FC *p* < 0.1) variants. ∗*p* < 0.006 and ∗∗∗*p* < 0.00006.

We identified 709 CCVs (14.98%) at 140 risk signals that showed significant differential activity between alleles (FDR-adjusted *p* < 0.1), ranging from −0.85 to 1.38 log_2_ fold change (FC) (Figure 2B; Data S1). Most of these (93.65%) showed modest effects (<20% differential activity) consistent with common variants individually making a small contribution to overall risk. On average, there were five CCVs showing differential allelic activity per risk signal (range: 1–42). Stratified analysis confirmed that allele log_2_FC was consistent across forward and reverse orientation (Figure S2; Table S1).

CCVs showing significant enhancer activity were enriched for larger allelic differences (|allele FC| > 10%, *p* = 1.51 × 10^−8^; Figure 2C). These variants also showed significant enrichment for DHS (*p* = 3.19 × 10^−5^) and EP300 binding (*p* = 8.79 × 10^−4^) in T-47D cells (Figures 2D and 2E), supporting their regulatory impact (Figure S3).

When comparing the 709 differentially expressed variants with BCAC data, 5.4% were index variants (1.5-fold enrichment, *p* = 0.03), and 1.0% were single CCVs (2-fold enrichment, *p* = 0.11). We also validated six variants that are the subject of single-locus studies (Table S2) and noted four additional variants that displayed strong enhancer activity but no allelic differences, making it possible that some previous studies prioritized enhancer presence over functional impact *per se*.

Variant prioritization was performed by filtering for those with |log_2_FC| > 0.07, excluding promoter regions, and selecting variants with significant enhancer activity and relevant epigenetic marks (Figure 3A; Table S3; full dataset in Data S1). Potential target genes were predicted based on those estimated by Fachal et al.,^1^ nearest transcription start site (TSS), expression quantitative trait loci (eQTLs) in “Breast-Mammary Tissue” reported by the Genotype-Tissue Expression (GTEx) Project, and promoter capture Hi-C (pCHi-C) data generated in T-47D^7^ (Table S3; Data S1). Among the 23 prioritized candidates, rs7153397 at 14q32.11 exhibited a log_2_FC of 0.85 (80% increase in activity; *p* = 1.5 × 10^−56^), was validated using conventional reporter gene assays in two different cell lines (T-47D and MCF-7; Figure S4), and was selected for further analysis. This variant is in perfect LD with the regional index variant rs11341843 (D′ = 1.0, r^2^ = 1.0; odds ratio for ER+ breast cancer = 1.06, *p* = 1.4 × 10^−11^),^1^ which did not have a significant allelic difference (*p* = 0.53). rs7153397 also maps to a region marked by H3K27ac and binding of FOXA1, ESR1, and EP300, consistent with a putative regulatory mechanism underlying increased breast cancer risk (Figures 3B and 3C). The 14q32.11 locus is associated with ER+ disease specifically, with the risk (ALT) allele associated with higher levels of target gene expression (Table S4).

**Figure 3:**
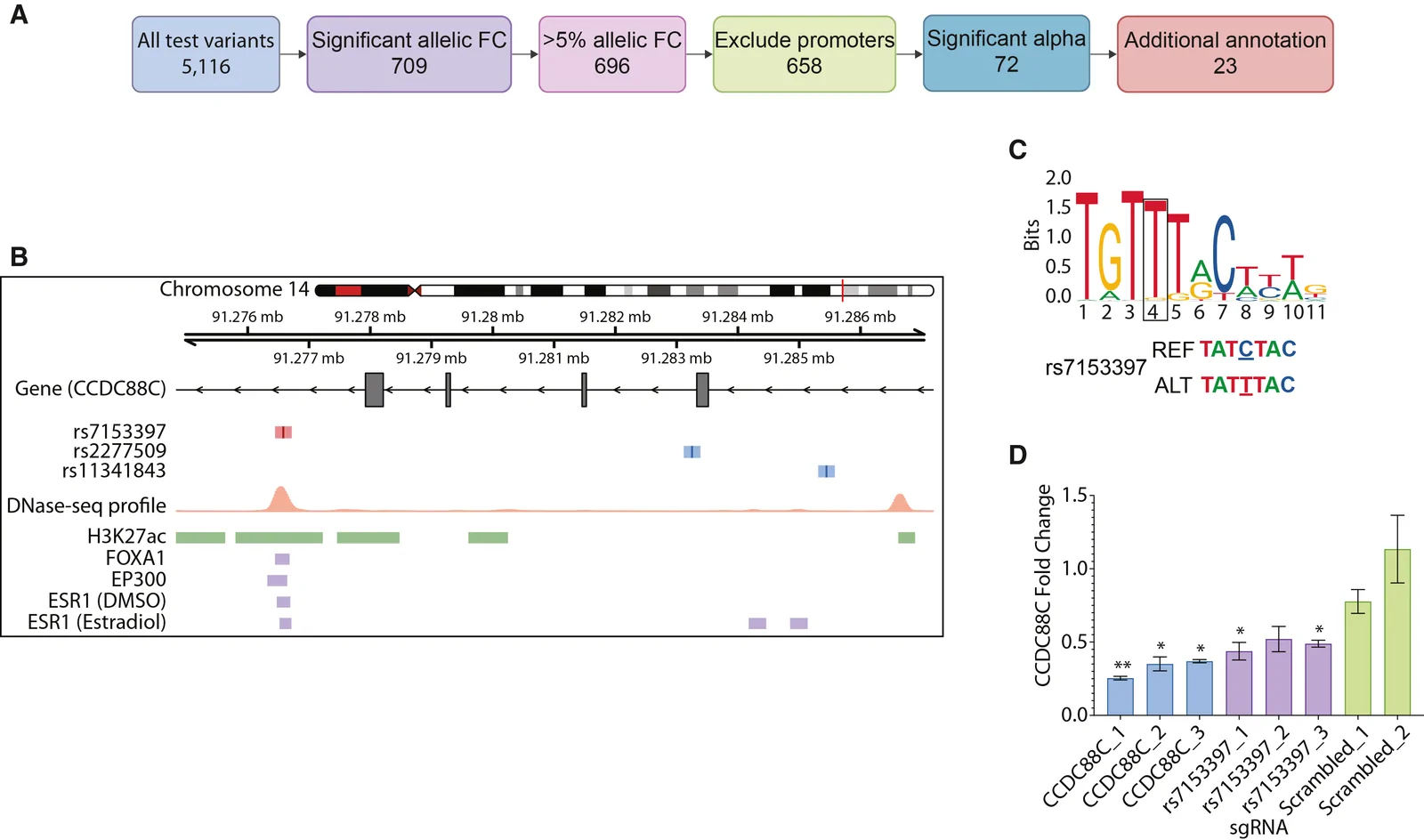
*CCDC88C* as a target gene for rs7153397 at 14q32.11 (A) Pipeline for selecting rs7153397 for functional follow-up. (B) Genomic region at 14q32.11 with test sequences (red: rs7153397, blue: other variants) and T-47D annotations. DNase sequencing profile, orange; H3K27ac, green; TF ChIP-seq, purple. (C) FOXA1 binding matrix with rs7153397 reference (REF) and alternative (ALT) sequences of rs7153397 shown below; the base corresponding to position 4 is underlined. (D) *CCDC88C* expression levels in T-47D cells following CRISPRi with sgRNAs targeting dCas9-KRAB to the *CCDC88C* promoter (CCDC88C_1, CCDC88C_2, and CCDC88C_3), rs7153397 variant (rs7153397_1, rs7153397_2, and rs7153397_3), or non-targeting negative controls (Scrambled_1 and Scrambled_2). Fold change was corrected for *GAPDH* and relative expression (compared to empty vector alone) was calculated for each sgRNA using the ΔΔCT method. Error bars correspond to standard error of the mean; *p* values are from *t* tests. A Bonferroni *p* value of 0.006 was considered significant. ∗*p* < 0.006 and ∗∗*p* < 0.0006.

To functionally validate the regulatory role of rs7153397 and its putative target gene, *CCDC88C*, CRISPR interference (CRISPRi) was performed in T-47D cells using dCas9-KRAB and single guide RNAs (sgRNAs) targeting the variant. Two rs7153397-specific sgRNAs significantly reduced *CCDC88C* expression (*p* = 0.002 and *p* = 0.004) (Figure 3D), with no effect on neighboring genes (*C14orf1159* or *PPP4R3A*; Figure S5), confirming *CCDC88C* as the likely target gene underlying the rs7153397-14q32.11 association. In The Cancer Genome Atlas (TCGA), *CCDC88C* expression was significantly elevated in ER+ and ER− breast tumors compared to normal breast tissue (*p* = 2.0 × 10^−23^ and *p* = 0.0002, respectively) and in both TCGA and the Sweden Cancerome Analysis Network-Breast (SCAN-B) cohorts, *CCDC88C* expression was significantly higher in ER+ compared to ER− tumors (*p* = 2.2 × 10^−15^ in TCGA; *p* = 1.4 × 10^−30^ in SCAN-B) (Figures 4A and 4B). Higher *CCDC88C* expression was also associated with improved survival in ER+ disease (hazard ratio [HR] = 0.53, *p* = 0.004 in TCGA; HR = 0.63, *p* = 1.9 × 10^−5^ in SCAN-B; Figures 4C and 4D) but not ER− disease (Figures 4E and 4F).

**Figure 4:**
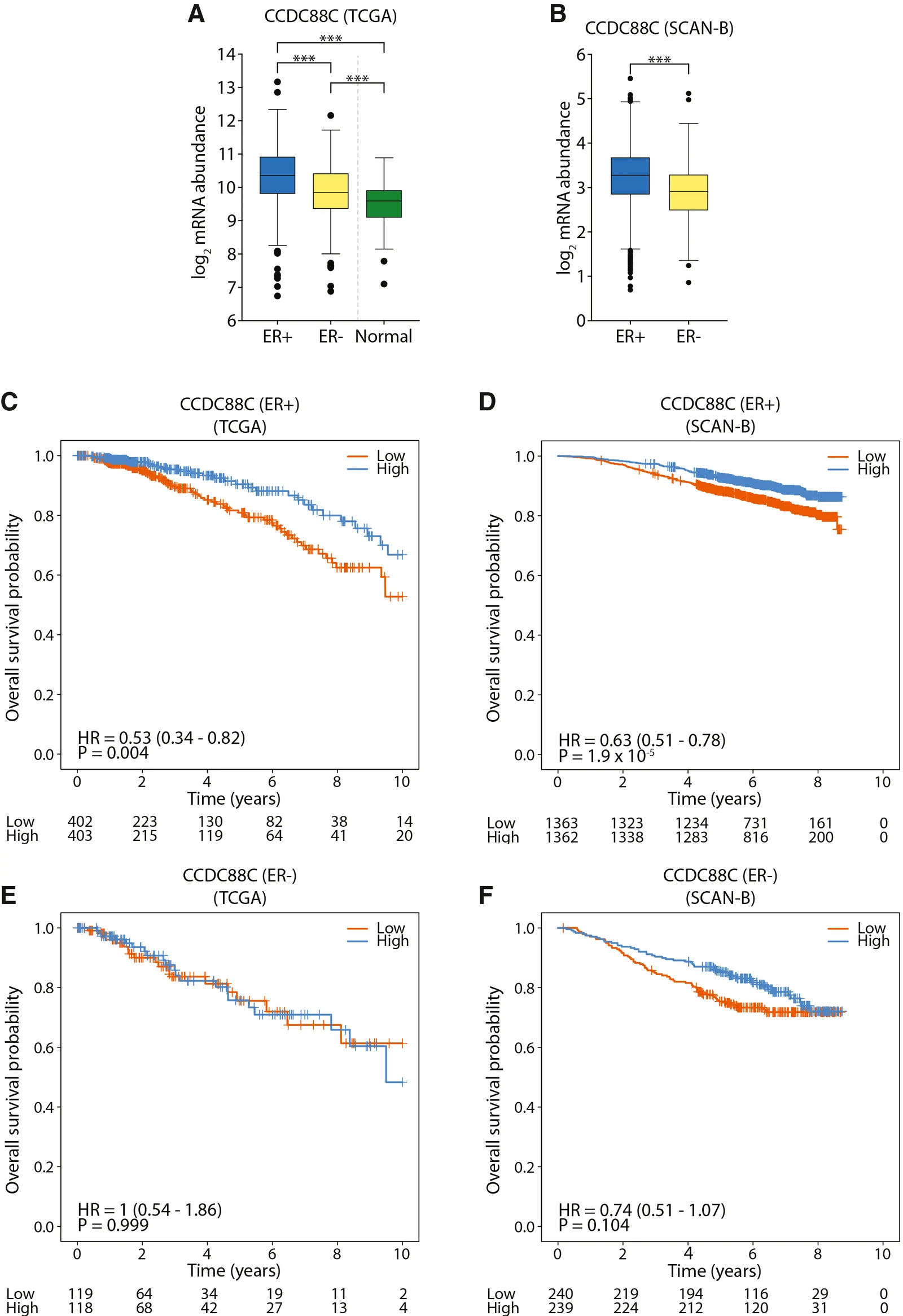
*CCDC88C* expression is associated with breast cancer subtype and survival (A and B) Boxplots of log_2_ abundance of *CCDC88C* mRNA in breast tissue stratified by ER status in data from (A) TCGA and (B) SCAN-B; ∗*p* < 0.01, ∗∗*p* < 0.001, and ∗∗∗*p* < 0.0001. The box represents the IQR, with whiskers 1.5× the IQR; the center line represents the median. (C–F) Kaplan-Meier plots of association between *CCDC88C* expression (above median expression [high] vs. below median expression [low]) in ER+ data from TCGA (C) and SCAN-B (D) and ER− data from TCGA (E) and SCAN-B (F).

## Discussion

We report the use of a high-throughput barcoded lentiMPRA to assess the functional effect of 5,116 CCVs identified by BCAC fine-mapping analysis.^1^ We identified 709 CCVs across 140 risk regions that show statistically significant allelic effects on reporter activity. Among these, we characterized rs7153397 at 14q32.11, identifying *CCDC88C* as its target gene and proposing a mechanism by which this variant might alter expression of *CCDC88C* to impact breast cancer risk.

Our choice of barcoded lentiMPRA to assay multiple variants was driven by its advantages over alternatives, such as self-transcribing active regulatory region sequencing (STARR-seq). Although STARR-seq offers simpler protocols and supports longer sequences,^8^ lentiMPRA’s use of multiple unique barcodes per candidate regulatory sequence (CRS) minimizes biases from RNA stability, thereby enabling more precise quantification of enhancer activity.^9^ Furthermore, lentiMPRA’s integration of reporter constructs into the host cell genome better recapitulates the *in vivo* chromatin context when compared to episomal assays, such as STARR-seq.^10^^,^^11^ These features thereby maximize the reliability of our functional readouts, especially for assessing regulatory activity in a chromatin-relevant setting.

Our study represents a comprehensive functional validation of BCAC-defined CCVs, which were identified using a combination of stepwise multinomial logistic regression and evaluated with the PAINTOR Bayesian model, which integrates genetic association, LD, and epigenomic data.^1^ Overall, we observed only a weak correlation between PAINTOR posterior probabilities (PPs) and both allelic activity (log_2_FC; Spearman’s *ρ* = 0.12) and enhancer strength (alpha; Spearman’s *ρ* = 0.08). However, among the eight variants predicted to be causal for ER+ breast cancer (PP > 0.8), five showed a significant difference in allelic activity, including rs7153397, with two also exhibiting enhancer activity. While four variants have been the subject of previous locus-specific analysis, the identification of rs7153397 as the functional basis of the 14q23 association has not been previously reported. Our findings therefore serve to highlight the complementary value of fine-mapping, high-throughput screening, and targeted follow-up in prioritizing potentially functional variants.

Despite the enrichment of variants with significant allelic effects in strong enhancers (FDR-adjusted *p* < 0.1), overlap was incomplete. We identified 222 CCVs lacking allelic differences that mapped to strong enhancers, suggesting these variants reside in regulatory regions but do not directly modulate enhancer activity, including four previously validated functional variants mapping to strong enhancers but lacking allelic effects. This discrepancy underscores the limitations of relying solely on epigenomic annotations, which may prioritize variants in active regulatory regions without confirming their functional impact. Additionally, 625 variants with significant allelic effects mapped to sequences without detectable enhancer activity. We acknowledge that this may reflect technical constraints, as the 270 bp oligonucleotides used may not fully capture the regulatory context of enhancers.^12^ Hence, these variants may reside in weak or context-dependent enhancers or require specific cellular or environmental cues for activity.

Our follow-up analysis of rs7153397 identified *CCDC88C* as the likely target gene at the 14q32.11 locus. *CCDC88C* encodes a regulator of non-canonical Wnt signaling via the PI3K-AKT pathway,^13^ which plays a role in mammary gland development^14^ and breast cancer progression^15^ but to date has not been implicated in early tumorigenesis. We demonstrated that rs7153397 resides within a putative FOXA1 binding site, and CRISPRi-mediated targeting of this site reduced *CCDC88C* expression, supporting a regulatory mechanism underlying its association with breast cancer risk. Consistent with the link between rs7153397 and ER+ breast cancer, *CCDC88C* expression was consistently increased in ER+ tumors, with higher expression being associated with better prognosis. While demonstrating that *CCDC88C* is expressed at higher levels in breast cancer compared to normal tissue does not prove that this expression originates from the cancer cells, nor provide evidence of causality, these data support *CCDC88C* upregulation contributing to ER+ breast cancer and influencing patient prognosis.

There are limitations to this study; specifically, the size of the CRS is restricted to 270 bp due to limits in large-scale oligonucleotide synthesis, the use of a generic lentiviral promoter may lead to false positive or false negative results, and post-transcriptional processes are not analyzed. In addition, our screen was carried out in a single ER+ breast cancer cell line. We selected T-47D on the grounds that it is ER+, and 85% of the signals reported by BCAC are associated with either ER+ disease predominantly or ER+ and ER− disease equally. Ideally, however, the screen should be repeated in additional ER+ breast cancer cell lines to assess the generalizability of our results and in ER− breast cancer cell line(s) and/or normal breast epithelial cells to investigate cell-type specificity. For validation of individual variants, we relied primarily on the literature, validating six variants at 2q35,^16^^,^^17^ 6q25.1,^18^ 9q31.2,^19^ and 10q26.13.^20^ However, we were able to validate rs7153397 at 14q32.11 using a conventional reporter gene assay and demonstrated that for this variant there was an association between the ALT allele and higher levels of transcription in an additional ER+ breast cancer cell line.

In conclusion, while this study alone cannot identify causal variants and target genes at the 196 breast cancer risk signals reported by Fachal et al.,^1^ it defines a functionally annotated subset of breast cancer risk variants that modulate enhancer activity, providing a refined list of high-priority CCVs for mechanistic follow-up. Unlike epigenomic annotations alone, our approach pinpoints variants with demonstrable regulatory effects, offering deeper insight into the functional architecture of breast cancer susceptibility.

## Data and code availability

All datasets generated during this study are provided in Data S1, and all analyses were performed using publicly available libraries, which are described in the supplemental material and methods.

## Acknowledgments

This work was funded by Program Funding to the Breast Cancer Now Toby Robins Research Centre, awarded to O.F. and S.H. This work was also supported by funding from 10.13039/501100000289Cancer Research UK (C1298/A2551) to R.S.H. This work represents independent research supported by the National Institute for Health Research (10.13039/501100000272NIHR) Biomedical Research Centre at The Royal Marsden NHS Foundation Trust and the Institute of Cancer Research, London. The views expressed are those of the authors and not necessarily those of the NIHR or the Department of Health and Social Care. We thank the patients with breast cancer who contributed to TCGA and SCAN-B. The results shown here are in whole or part based on data generated by TCGA research network (https://www.cancer.gov/tcga). We acknowledge the Genomics facility at the ICR for providing expert sequencing support of the samples. The GTEx Project was supported by the Common Fund of the Office of the Director of the 10.13039/100000002National Institutes of Health and by the NCI, 10.13039/100000051NHGRI, 10.13039/100000050NHLBI, NIDA, 10.13039/100000025NIMH, and 10.13039/100000065NINDS. The data used for the analyses described in this manuscript were obtained from the GTEx API on April 20, 2026. pLS-SceI was gifted by Nadav Ahituv (Addgene plasmid #137725; http://n2t.net/addgene:137725; RRID:Addgene_137725), psPAX2 was gifted by Didier Trono (Addgene plasmid #12260; http://n2t.net/addgene:12260; RRID:Addgene_12260), pMD2.G was gifted by Didier Trono (Addgene plasmid #12259; http://n2t.net/addgene:12259; RRID:Addgene_12259), pKLV2-U6gRNA5(BbsI)-PGKpuro2AZsG-W was a gift from Kosuke Yusa (Addgene plasmid #67975; http://n2t.net/addgene:67975; RRID:Addgene_67975), and Lenti-dCas9-KRAB-blast was gifted by Gary Hon (Addgene plasmid #89567; http://n2t.net/addgene:89567; RRID:Addgene_89567).

## Author contributions

Conceptualization, O.F., N.J., and S.H.; methodology, K.M., O.F., N.J., and S.H.; investigation, K.M., H.K., A.G., K.T., S.S., R.F., and E.L.; formal analysis, K.M., H.K., O.F., S.H., Y.L., J.B.S., M.W., P.L., R.S.H., and N.O.; supervision, O.F., N.J., and S.H.; writing – original draft, K.M., O.F., R.S.H., H.K., N.J., and S.H.; writing – review & editing, all authors.

## Declaration of interests

The authors declare no competing interests.

## Web resources

Addgene (Lenti-dCas9-KRAB-blast), http://n2t.net/addgene:89567
Addgene (pKLV2-U6gRNA5(Bbsl)-PGKpuro2AZsG-W), http://n2t.net/addgene:67975
Addgene (pLS-Scel), http://n2t.net/addgene:137725
Addgene (pMD2.G), http://n2t.net/addgene:12259
Addgene (psPAX2), http://n2t.net/addgene:12260
TCGA, https://www.cancer.gov/tcga

## Supplemental information

Document S2. Tables S1–S5 and Data S1

Document S3. Article plus supplemental information

## References

[1] Fachal L., Aschard H., Beesley J., Barnes D.R., Allen J., Kar S., Pooley K.A., Dennis J., Michailidou K., Turman C. (2020). Fine-mapping of 150 breast cancer risk regions identifies 191 likely target genes. Nat. Genet..

[2] Udler M.S., Tyrer J., Easton D.F. (2010). Evaluating the power to discriminate between highly correlated SNPs in genetic association studies. Genet. Epidemiol..

[3] Gordon M.G., Inoue F., Martin B., Schubach M., Agarwal V., Whalen S., Feng S., Zhao J., Ashuach T., Ziffra R. (2020). lentiMPRA and MPRAflow for high-throughput functional characterization of gene regulatory elements. Nat. Protoc..

[4] Eeckhoute J., Keeton E.K., Lupien M., Krum S.A., Carroll J.S., Brown M. (2007). Positive cross-regulatory loop ties GATA-3 to estrogen receptor alpha expression in breast cancer. Cancer Res..

[5] Hevir N., Trošt N., Debeljak N., Rižner T.L. (2011). Expression of estrogen and progesterone receptors and estrogen metabolizing enzymes in different breast cancer cell lines. Chem. Biol. Interact..

[6] Ashuach T., Fischer D.S., Kreimer A., Ahituv N., Theis F.J., Yosef N. (2019). MPRAnalyze: statistical framework for massively parallel reporter assays. Genome Biol..

[7] Zvereva A. (2026). Functional annotation of breast cancer risk loci implicates perturbation of *FILIP1L* expression in mammary fibroblasts in influencing breast cancer risk. medRxiv.

[8] Lee D., Shi M., Moran J., Wall M., Zhang J., Liu J., Fitzgerald D., Kyono Y., Ma L., White K.P., Gerstein M. (2020). STARRPeaker: uniform processing and accurate identification of STARR-seq active regions. Genome Biol..

[9] Inoue F., Ahituv N. (2015). Decoding enhancers using massively parallel reporter assays. Genomics.

[10] Inoue F., Kircher M., Martin B., Cooper G.M., Witten D.M., McManus M.T., Ahituv N., Shendure J. (2017). A systematic comparison reveals substantial differences in chromosomal versus episomal encoding of enhancer activity. Genome Res..

[11] Kreimer A., Yan Z., Ahituv N., Yosef N. (2019). Meta-analysis of massively parallel reporter assays enables prediction of regulatory function across cell types. Hum. Mutat..

[12] Parker S.C.J., Stitzel M.L., Taylor D.L., Orozco J.M., Erdos M.R., Akiyama J.A., van Bueren K.L., Chines P.S., Narisu N., NISC Comparative Sequencing Program (2013). Chromatin stretch enhancer states drive cell-specific gene regulation and harbor human disease risk variants. Proc. Natl. Acad. Sci. USA.

[13] Aznar N., Sun N., Dunkel Y., Ear J., Buschman M.D., Ghosh P. (2017). A Daple-Akt feed-forward loop enhances noncanonical Wnt signals by compartmentalizing beta-catenin. Mol. Biol. Cell.

[14] Rosen J.M., Roarty K. (2014). Paracrine signaling in mammary gland development: what can we learn about intratumoral heterogeneity?. Breast Cancer Res..

[15] Lindvall C., Evans N.C., Zylstra C.R., Li Y., Alexander C.M., Williams B.O. (2006). The Wnt signaling receptor Lrp5 is required for mammary ductal stem cell activity and Wnt1-induced tumorigenesis. J. Biol. Chem..

[16] Ghoussaini M., Edwards S.L., Michailidou K., Nord S., Cowper-Sal Lari R., Desai K., Kar S., Hillman K.M., Kaufmann S., Glubb D.M. (2014). Evidence that breast cancer risk at the 2q35 locus is mediated through IGFBP5 regulation. Nat. Commun..

[17] Dryden N.H., Broome L.R., Dudbridge F., Johnson N., Orr N., Schoenfelder S., Nagano T., Andrews S., Wingett S., Kozarewa I. (2014). Unbiased analysis of potential targets of breast cancer susceptibility loci by Capture Hi-C. Genome Res..

[18] Dunning A.M., Michailidou K., Kuchenbaecker K.B., Thompson D., French J.D., Beesley J., Healey C.S., Kar S., Pooley K.A., Lopez-Knowles E. (2016). Breast cancer risk variants at 6q25 display different phenotype associations and regulate ESR1, RMND1 and CCDC170. Nat. Genet..

[19] Orr N., Dudbridge F., Dryden N., Maguire S., Novo D., Perrakis E., Johnson N., Ghoussaini M., Hopper J.L., Southey M.C. (2015). Fine-mapping identifies two additional breast cancer susceptibility loci at 9q31.2. Hum. Mol. Genet..

[20] Meyer K.B., O'Reilly M., Michailidou K., Carlebur S., Edwards S.L., French J.D., Prathalingham R., Dennis J., Bolla M.K., Wang Q. (2013). Fine-scale mapping of the FGFR2 breast cancer risk locus: putative functional variants differentially bind FOXA1 and E2F1. Am. J. Hum. Genet..

