# Supplementary material for: Massively parallel functional profiling prioritizes breast cancer risk variants and implicates CCDC88C in ER-positive breast cancer
Source: HGG Adv. 2026 Sep 17;7(4):100674. doi: 10.1016/j.xhgg.2026.100674 (PMC13657374; doi:10.1016/j.xhgg.2026.100674)
Supplement: Supplementary file 1 [file mmc1.pdf]

HGGA, Volume *7*

Supplemental information

Massively parallel functional profiling prioritizes breast cancer risk variants and implicates *CCDC88C* in ER-positive breast cancer

Kate Mackie, Harriet Kemp, Andrea Gunnell, James B. Studd, Molly Went, Philip Law, Katarzyna Tomczyk, Rafaela Ferreira, Ernesto Lopez, Selin Sevgi, Yunjiao Lu, Alisa Zvereva, Nick Orr, Richard S. Houlston, Nichola Johnson, Olivia Fletcher, and Syed Haider

Supplemental Table of Contents

SUPPLEMENTAL FIGURES **2**

Figure S1. Replicate correlations. 2

Figure S2. Allelic log_2FC analysis. 3

Figure S3. Additional enrichment. 4

Figure S4. Reporter gene assay validation. 5

Figure S5. CRISPRi gene expression. 6

Figure S6. dCas9 western blot. 7

MATERIALS AND METHODS **8**

REFERENCES **20**

SUPPLEMENTAL FIGURES

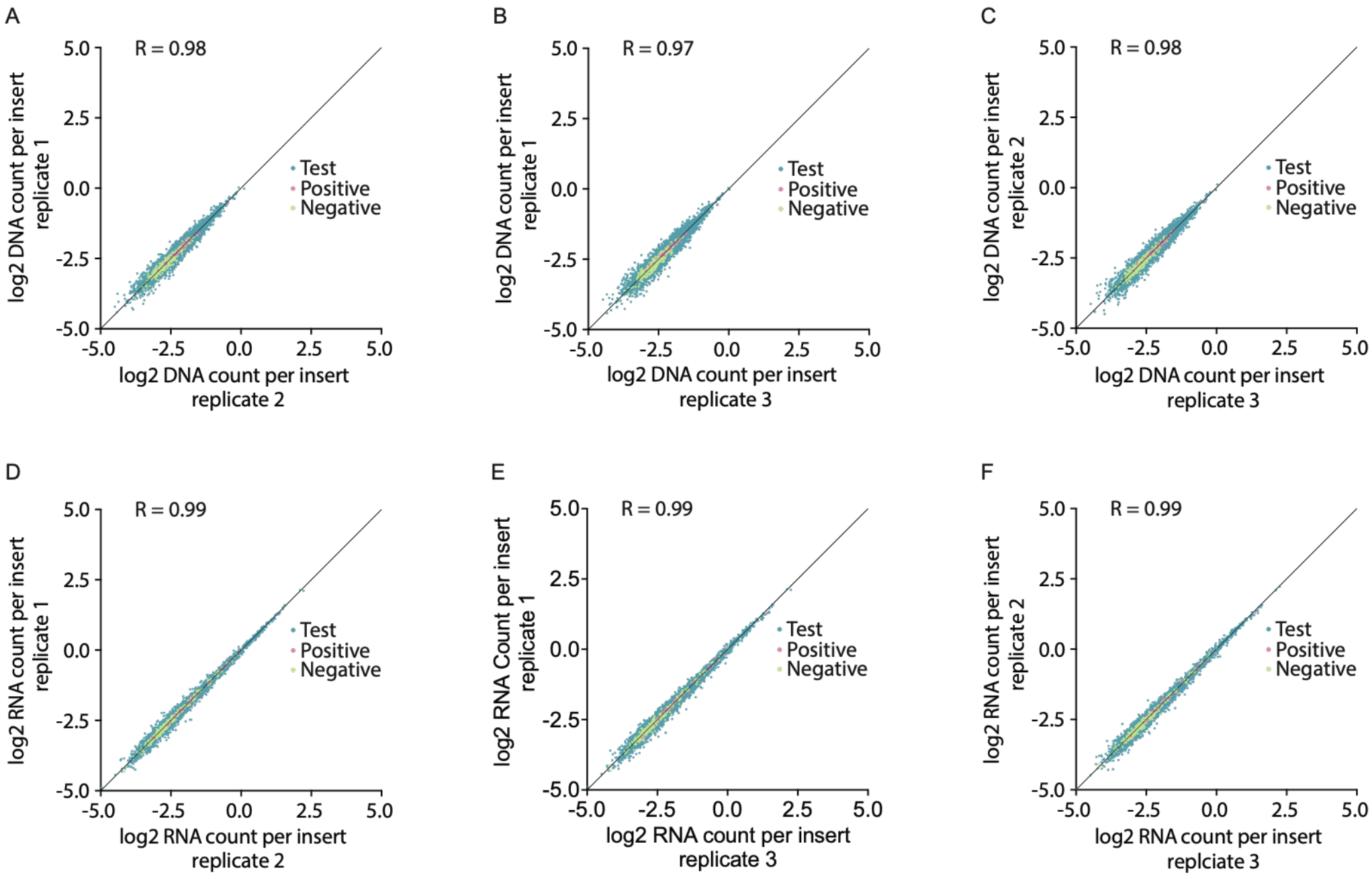

Figure S1. Replicate correlations.

A-C, Correlation of log_2 normalized DNA count per insert of **(A)** replicate 1 against replicate 2, **(B)** replicate 1 against replicate 3, **(C)** replicate 2 against replicate 3.

D-F, Correlation of log_2 normalized RNA count per insert of **(D)** replicate 1 against replicate 2, **(E)** replicate 1 against replicate 3, **(F)** replicate 2 against replicate 3.

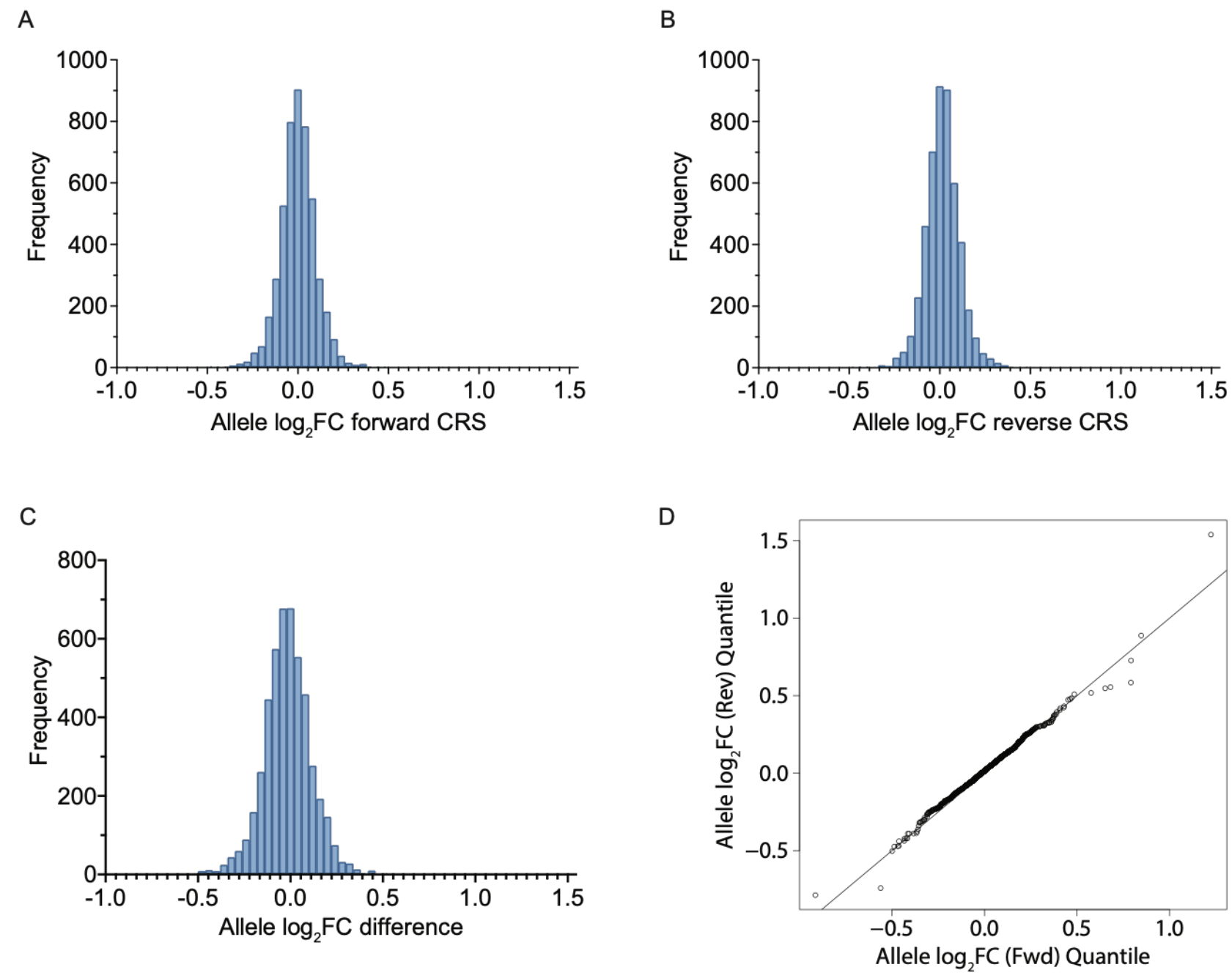

Figure S2. Allelic log_2FC analysis.

A and B, Frequency of allele log_2FC from stratified analysis testing **(A)** forward CRS and **(B)** reverse CRS independently.

C, Frequency of the difference between output from stratified analysis of allele log_2FC on the forward strand and allele log_2FC on the reverse strand.

D, QQ plot of the quantiles of allele log_2FC on the forward strand against quantiles of allele log_2FC on the reverse strand.

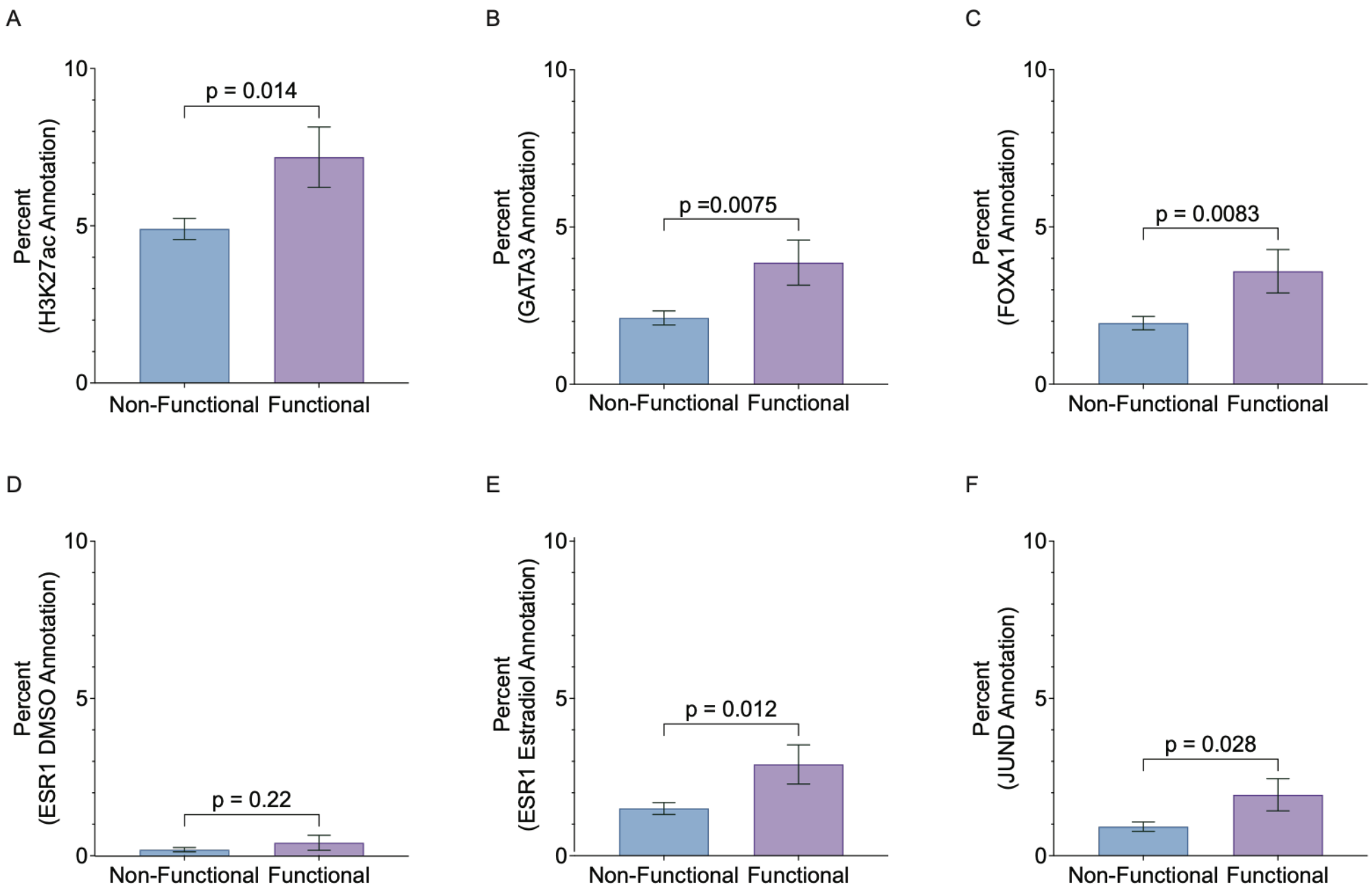

Figure S3. Additional enrichment.

A-F, Percentage of sequences with H3K27ac **(A),** *GATA3* **(B)** *FOXA1* **(C),** *ESR1* (DMSO) **(D)** *ESR1* (estradiol) **(E)** and *JUND* **(F)** annotations comparing non-functional (allele log_2FC $P > 0.1$) and functional variants (allele log_2FC $P < 0.1$). Error bars denote standard error of the mean; *P* values from two-sided Fisher's exact test, and a Bonferroni correction was applied to account for multiple testing with a *P* value of 0.006 considered as significant. All non-significant.

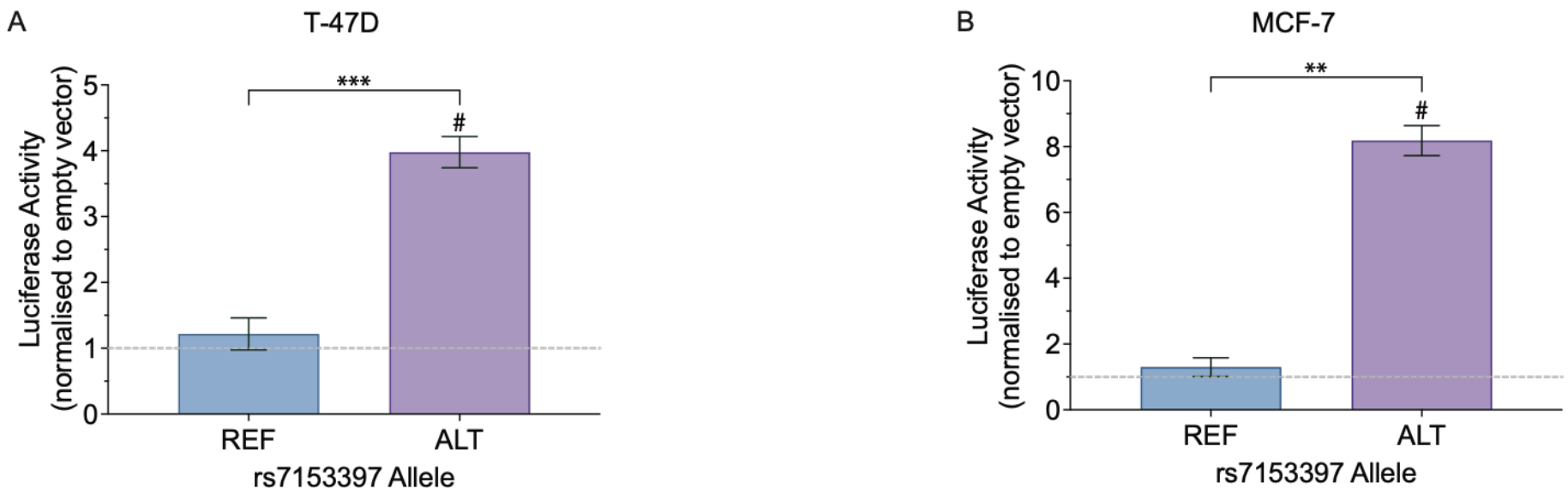

Figure S4. Reporter gene assay validation.

A and B, Luciferase activity in T-47D **(A)** and MCF-7 **(B)** cells following transient transfection of rs7153397 REF and ALT constructs, normalised to pGL4.23 vector with minimal promoter only (dashed line). Error bars denote standard error of the mean; *P* values were determined by *t*-tests and a Bonferroni correction was applied to account for multiple testing. Comparing each construct to pGL4.23 vector with minimal promoter, # $P < 0.025$; comparing ALT to REF constructs, ** $P < 0.0025$, *** $P < 0.00025$.

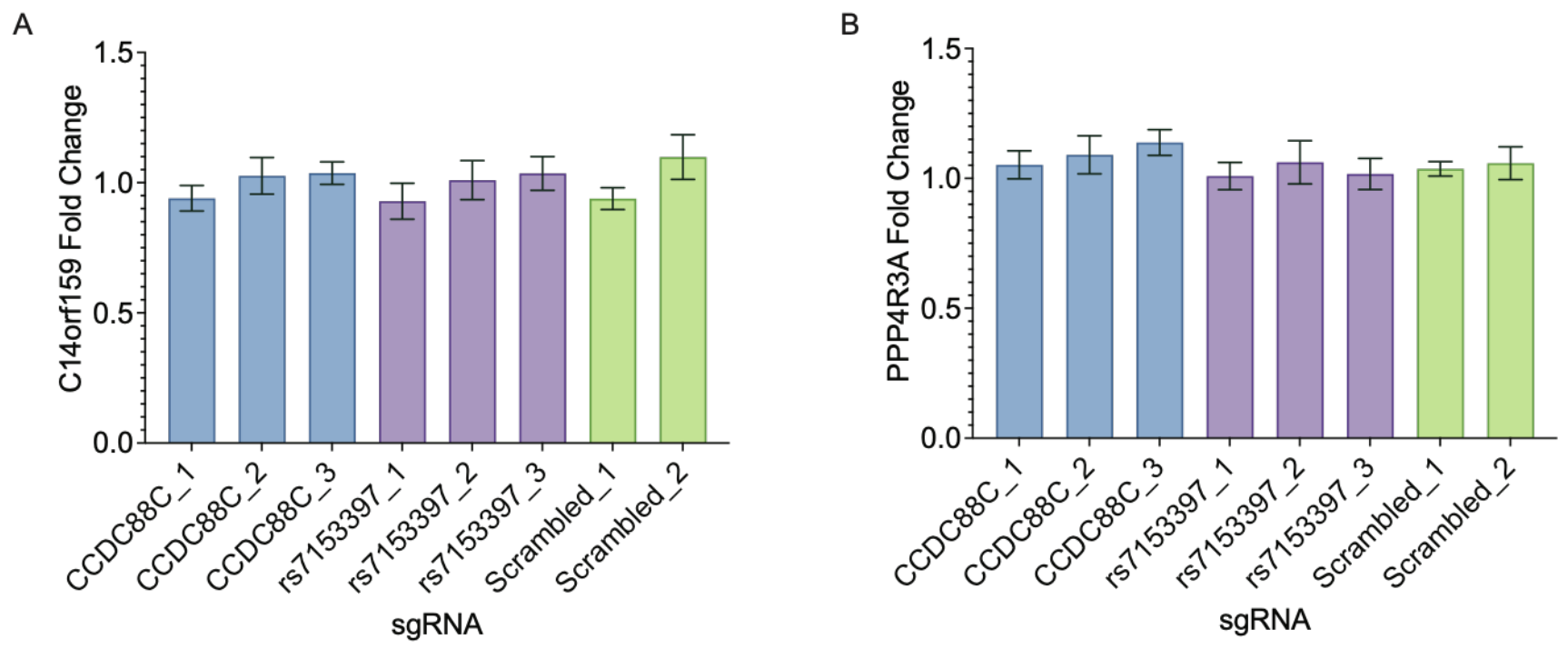

Figure S5. CRISPRi gene expression.

A and B, Expression levels of *C14orf159* **(A)** and *PPP4R3A* **(B)** in T-47D cells following CRISPRi with sgRNAs targeting dCas9-KRAB to the *CCDC88C* promoter (CCDC88C_1, CCDC88C_2, CCDC88C_3), rs7153397 variant (rs7153397_1, rs7153397_2, rs7153397_3) or non-targeting negative controls (Scrambled_1, Scrambled_2). Fold change was corrected for *GAPDH* and relative expression (compared to empty vector alone) was calculated for each sgRNA using the ΔΔCT method. Error bars denote standard error of the mean; *P* values from *t*-tests and a Bonferroni correction was applied to account for multiple testing with a *P* value of 0.006 considered as significant. All non-significant.

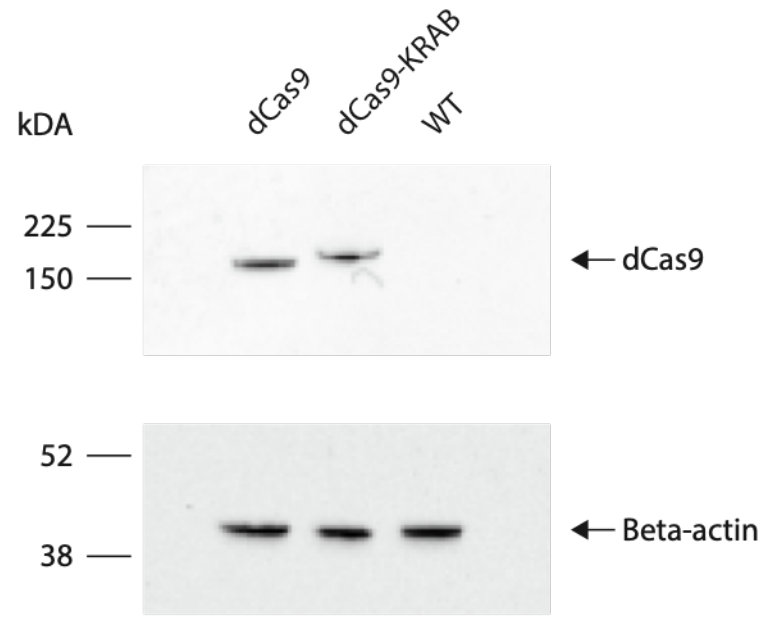

Figure S6. dCas9 western blot.

Western blot of whole cell lysates from T-47D cells that have been modified to express dCas9 (lane 1, positive control), dCas9-KRAB (lane 2) and T-47D wildtype cells (lane 3, negative control). Blots were probed with an anti-dCas9 antibody. As a loading control, blots were probed with an antibody against beta-actin.

MATERIALS AND METHODS

Experimental design

We screened 5,116 breast cancer associated credible causal variants (CCVs), which were reported by the Breast Cancer Association Consortium (BCAC) fine mapping analysis[1], using a lentivirus-based massive parallel reporter assay (lentiMPRA)[2] in T-47D cells to identify variants with differential regulatory activity. We designed 270 bp oligonucleotides centred on reference (REF) or alternative (ALT) alleles, packaged them into a lentivirus library and infected T-47D cells. We sequenced DNA and RNA to assess regulatory activity, using available datasets to annotate our library. We followed up on rs7153397 with CRISPR interference (CRISPRi) and analysed *CCDC88C* expression in breast cancer cohorts.

CCV selection and coordinate conversion

We selected 5,116 CCVs reported by BCAC [1], excluding 2,277 in strong LD with a copy number variant (index variant rs62070949) and one withdrawn from the hg38 version of the human reference genome (rs72127681).

We converted the genomic coordinates of CCVs from human genome build hg19 to hg38 using SNP rs IDs as input against dbSNP version 151 and dbSNP version 155 using BCFtools version 1.11 'view' function, within R 3.6.0 environment. The hg38-equivalent IDs and coordinates for COSMIC variants and historically merged SNPs were inputted manually (rs1491361031, rs36094352, rs78105048, rs146915990, rs587714804, rs143207801, rs114312440, rs559449702, rs142901261, rs1353154669, rs533235186, rs111769614, rs528673091, rs8039, rs142118767, COSM3756504). Where a variant did not have an associated rs ID in the BCAC hg19 list, position was queried using liftOver version 20180306.

Oligonucleotide design

We designed 20,878 oligonucleotides of 270 bp centred on reference (REF) or alternative (ALT) alleles in forward and reverse orientations, with 15 bp adaptors[2]. We extracted the up- and down-stream nucleotide sequences surrounding each CCV in the hg38 human reference genome, using a custom R script. For SNPs, 135 bp downstream and 134 bp upstream of the CCV was selected, and for insertion/deletion polymorphisms, flanking sequence length was adjusted depending on variant length, such that flanking sequence remained consistent for each pair of reference and alternative alleles. Adaptors were added in both orientations to create forward and reverse sequences for each CCV. The library included 36 positive controls near *GATA3* and *PGR* and 400 negative controls from scrambled sequences. Scrambled negative control sequences were generated by randomly sampling nucleotides to match the length of test sequences, excluding homopolymers and the SceI restriction enzyme recognition site. Each negative sequence was paired with a single-nucleotide variant at a fixed position to mimic REF and ALT alleles. Oligonucleotides were synthesised by Twist Bioscience.

Library generation

The oligonucleotide pool was amplified via two-stage PCR. A 5-cycle PCR added minimal promoter and adaptor sequences downstream of the CRS, and the second 15-cycle PCR added a 15 bp random barcode sequence downstream of the first-round fragment. Amplified sequences were ligated into a pLS-SceI vector (Addgene #137725[2]) using NEBuilder HiFi DNA Assembly Master Mix (NEB) and electroporated into 10-beta electrocompetent cells (NEB, C3020) using an Eppendorf Eporator. Colonies were grown overnight on ampicillin plates, supplemented with 100 µl/plate of 100mg/ml carbenicillin (Teknova). The plasmid library was purified with QIAgen Plasmid Plus Midi Kit (Qiagen, 12943). 3.13 million colonies were harvested to ensure each oligonucleotide sequence was associated with around 150 barcodes. The plasmid library was sequenced to determine barcode sequences and their CRS association. CRS-barcode fragments were amplified to add P5 and P7 flowcell sequences

upstream and downstream, respectively. Sequencing was carried out using NextSeq P1 with 300 cycles and custom primers [2].

Cell line culture

T-47D cells (HTB-133, ATCC) were cultured in RPMI-1640 (Gibco) supplemented with 10% foetal bovine serum (FBS; Gibco) and 10 μg/ml human insulin (Sigma) at 37°C, 5% CO_2. 293T cells (CRL-3216, ATCC) and MCF-7 cells (HTB-22, ATCC) were cultured in DMEM (Gibco) supplemented with 10% FBS at 37°C, 5% CO_2. Cells were routinely tested for Mycoplasma contamination.

Lentivirus packaging and titration

Lentivirus packaging and titration was carried out as described previously[2]. 293T cells were seeded at 1.1×10^7 cells per T225 flask and incubated for 48 hours, four flasks were used for the 3 million barcode library. Cells were transfected with 10 μg/flask plasmid library, 6.5 μg/flask psPAX2 (Addgene #12260) and 3.5 μg/flask pMD2.G (Addgene #12259) using EndoFectin (GeneCopoeia) and incubated for 8.5 hours. Cell media was refreshed with 5% FBS and 1x ViralBoost reagent (Alstem) and cultured for a further 40 hours. Supernatant was filtered to isolate lentivirus and then concentrated using Lenti-X concentrator reagent (Takara).

T-47D cells were seeded for lentivirus titration at 1.75×10^5 cells per well of a 24-well plate and incubated for 24 hours. Lentivirus (0, 1, 2, 4, 8, 16, 32 or 64 μl) was added with Polybrene (Sigma-Aldrich) at a final concentration of 8 μg/ml and cells were incubated for 24 hours. The next day fresh media was added without Polybrene, and the cells were incubated for a further 2 days. Cells were washed three times with DPBS, and genomic DNA was extracted using Wizard SV genomic DNA purification kit (Promega). Multiplicity of infection (MOI) was determined as the ratio of viral DNA to genomic DNA, accounting for relative plasmid

backbone DNA, which represents non-integrated virus. This was measured by quantitative PCR (qPCR) using SYBR Green Mastermix (Applied Biosystems).

Lentiviral infection and DNA and RNA sequencing

Lentiviral infection and subsequent DNA and RNA sequencing was carried out as described previously[2] in triplicate, to obtain three biological replicates. T-47D cells were seeded at 3.7×10^6 cells per replicate, to allow for 90 integrations per barcode, in a 15 cm plate and incubated for 24 hours. Cells were infected with the lentiviral library, at an estimated MOI of 80, and Polybrene (Sigma-Aldrich) was added at 8 μg/ml final concentration. The next day fresh media was added without Polybrene, and cells were cultured for two more days. Cells were washed three times with DPBS, and genomic DNA and total RNA were extracted from cells using the AllPrep DNA/RNA Mini Kit (Qiagen) and RNase-Free DNase Set (Qiagen). RNA samples were DNase treated, using TURBO DNA-free Kit (Invitrogen) and reverse transcribed to add a 16 bp UMI and P7 flowcell sequence downstream of the barcode. We continue to refer to these samples as RNA in subsequent steps to distinguish them from genomic DNA samples. Library prep was carried out following the protocol for a high-complexity library[2], with a 3-cycle first round PCR reaction to add the P5 flowcell sequence and sample index sequence upstream and 16 bp UMI and P7 flowcell sequence downstream of the barcode. The second round PCR reaction was carried out with 17 cycles for DNA samples and 15 cycles for RNA samples. Sequencing was completed on a NovaSeq 6000 using custom primers.

LentiMPRA data analysis

Sequences from both the association and DNA/RNA sequencing steps were output in fastq format. From the association step there were 108 million reads, with a length of 146 bp (CRS upstream and CRS downstream) or 15 bp (Barcode). The DNA/RNA step produced between 56.9 and 88.7 million reads per replicate for DNA, and between 144.8 and 283.6 million reads per replicate for RNA, with a length of 15 bp (barcode forward and barcode reverse) or 16 bp (UMI).

Raw fastq sequences from the association step were input into a custom shell script to add 15 bp adaptors to each end of the sequences. The association.nf function of MPRAflow[2] was ran in Nextflow version 20.10.0 with the cigar flag specified as 300M and mapq flag set to 30, producing a pickle file as output. This output file was used to obtain barcode counts with MPRAflow Pipeline Version 2.3.5; CRS were removed if they were associated with less than 20 unique barcodes.

Raw fastq sequences from the DNA/RNA count step were ran with the count.nf function of MPRAflow[2] using Nextflow version 20.10.0 with the flags thresh 20, bc-length 15, umi-length 16 and merge-intersect TRUE. The run was repeated with the --mpranalyze flag to produce input for MPRAnlayze[3]. This input was formatted using a custom script within R (v4.4.1) to exclude CRS with no barcodes, specify positive and negative controls, remove CRS if either DNA or RNA data was missing, and remove variants that did not have data for all four CRS types (i.e. fwd-REF, fwd-ALT, rev-REF, rev-ALT). Barcodes were limited to a maximum of 200 per CRS, and the resulting matrices were used to create an MPRAObject[3]. MPRAnalyze version 1.9.1 was used for this and subsequent MPRAnalyze analysis.

The library size of the MPRAObject was normalised using the estimateDepthFactors depth estimator totsum. Subsequent quantification analysis was carried out without parallelisation using the analyzeQuantification, getAlpha and testEmpririical functions of MPRAnalyze. The analyzeComparative function of MPRAnalyze was ran to compare differential allelic activity[3].

H3K27ac CUT&Tag data generation

T-47D cells were prepared for CUT&Tag following the protocol from Active Motif (https://www.activemotif.com/catalog/1320/cut-tag-service). Briefly, adherent cells were washed in 1X PBS and collected in 1X Enzyme Free Cell Dissociation Solution Hank's Based (Sigma) using a cell scraper at room temperature. Cells were counted using a Countess II

Automated Cell Counter (Thermo Fisher), pelleted at 500 xg for 5 minutes at 4°C, and resuspended in ice-cold preservation solution (50% FBS/40% growth media/10% DMSO (Sigma)) at a concentration of 400 cells/µl. 500 µl (200,000 cells) was transferred to a 1.5 ml tube on ice and cells were gradually frozen to -70°C in a styrofoam container. Cells were shipped on dry ice to Active Motif for H3K27ac CUT&Tag data generation. Briefly, cells were incubated overnight with concanavalin A beads and 1 µl of the primary anti-H3K27ac antibody per reaction (#39135, Active Motif). After incubation with the secondary anti-rabbit antibody (1:100), cells were washed and tagmentation was performed at 37°C using protein-A-Tn5. Tagmentation was halted by the addition of EDTA, SDS and proteinase K at 55°C, after which DNA extraction and ethanol purification was performed, followed by PCR amplification and barcoding (see Active Motif CUT&Tag kit, #53160 for recommended conditions and indexes). Following SPRI bead cleanup (Beckman Coulter), the resulting DNA libraries were quantified and sequenced on an Illumina NextSeq 550 (8 million reads, 38 bp PE reads).

H3K27ac CUT&Tag data analysis

Reads were aligned using the BWA algorithm (mem mode; default settings)[4]. Duplicate reads were removed, and only reads that mapped uniquely (mapping quality ≥ 1) and as matched pairs were used for further analysis. Peaks were identified using the MACS 3.0.0 algorithm at a cutoff FDR corrected *P*-value of 0.05, without control file, and with the –nomodel option. Peaks that were on the ENCODE blacklist of known false ChIP-seq peaks were removed. Signal maps and peak locations were used as input data to Active Motif's proprietary analysis program, which creates output containing information on sample comparison, peak locations and gene annotations. Consensus peaks per cell type were defined as the maximum coordinates of peaks present by intersect in both replicates.

Oligonucleotide library annotation

We annotated the oligonucleotide library with available data using custom scripts in R (v4.4.1). We used direct intersect with Bedtools (v2.29.2) to annotate each CCV against gene

promoters, H3K27ac peaks, DHS and TF ChIP-seq peaks. Gene promoters were defined as 1 kb downstream and 500 bp upstream of a transcription start site (TSS) in the Gencode v46 basic gene annotation table (UCSC table wgEncodeGencodeBasicV46[5]). T-47D TF ChIP-seq and DHS peaks were downloaded from ENCODE 4 hg38 as narrow peaks without further processing: ENCFF574HSR (*GATA3*), ENCFF420MLJ (*FOXA1*), ENCFF433NIE (*ESR1* treated with DMSO) ENCFF597QIF (*ESR1* treated with estradiol), ENCFF387ZTS (*EP300*), ENCFF177MVH (*JUND*) and ENCFF592NQU (DHS). CCVs were assigned to their nearest Gencode v46 basic TSS using R package: genomation (v1.38.0). Previously defined candidate target genes were also annotated from Fachal, et al. [1] (level 1 predicted target genes from Supplementary Table 6a; predicted target genes with largest confidence level for each variant from Table 1).

CRISPR-based perturbation (CRISPRi)

Single guide RNAs (sgRNAs) were designed against target sequences for repression, using CHOPCHOP (http://chopchop.cbu.uib.no). Target sequences were selected based on their proximity to variants or gene promoters of interest, and specificity scores. Non-targeting scrambled sgRNAs from Gasperini, et al. [6] were used as negative controls (**Supplementary Table 5**). Complementary single-stranded DNA oligonucleotide pairs were designed with 5'-CACC (top strand) and 5'-AAAC (bottom strand) overhangs to facilitate cloning. If absent, a G nucleotide was substituted at the first base of the target sequence to improve transcription from the U6 promoter. DNA oligonucleotides were synthesised by IDT and cloning was performed as previously described[7]. Briefly, top and bottom strand sgRNA pairs were phosphorylated and annealed in a 10 µl PCR reaction, then diluted to 7.1 fmol/µl in EB Buffer (Qiagen). The guide expression vector pKLV2-U6gRNA5(BbsI)-PGKpuro2AZsG-W (Addgene #67975[8]) was linearised in a 50 µl reaction using 2 µg with 30 U BbsI (NEB) and 1x NEBuffer r2.1 (NEB). Annealed oligonucleotides were ligated into gel purified linear vector in a 10 µl reaction using 2 µl oligonucleotides, 20 ng linear vector, 20 U T4 ligase (NEB) and 1x ligase buffer (NEB), which was incubated at 16°C for a minimum of 5 hours. The ligation mixture was

transformed using 5 µl with 50 µl chemically competent bacterial cells, and successful cloning was confirmed by Sanger sequencing (GENEWIZ, Azenta Life Sciences).

Cloned guide expression vectors were packaged into lentivirus in 293T cells. For each vector, $2x10^6$ 293T cells were seeded in a T25 flask and incubated overnight. Cells were transfected with 500 ng/flask plasmid, 500 ng/flask psPAX2 (Addgene #12260) and 150 ng/flask pMD2.G (Addgene #12259) using 4 µl Lipofectamine 3000 (ThermoFisher Scientific), 2.5 µl P3000 reagent (ThermoFisher Scientific) and 125 µl OptiMEM (ThermoFisher Scientific) and cultured for 24 hours. Cell media was refreshed and cells cultured for a further 48 hours. Culture media containing lentivirus particles was collected and stored at 4°C. Fresh media was added to the flasks and cells were cultured for a further 24 hours. A second aliquot of media containing lentivirus particles was collected and added to the first. This was centrifuged at 1600 xg for 10 minutes at 4°C to pellet cellular debris. Supernatant was filtered through Millex-HV 0.45 µM PVDF filters (Millipore), concentrated with Lenti-X concentrator reagent (Takara) for 30 minutes at 4°C and centrifuged at 1500 xg for 45 minutes at 4°C. The pellet was resuspended in 1/10 of the original volume using DPBS and stored at 4°C before use.

T-47D cells stabling expressing dCas9-KRAB, for CRISPR interference (CRISPRi), had previously been derived from parental cell lines in the lab, by transduction with lentivirus. Lentivirus was packaged as described above, using Lenti-dCas9-KRAB-blast (Addgene #89567[9]) rather than sgRNA containing vectors. Parental cell lines were seeded in a T75 flask, cultured to 50% confluency and 500 µl lentiviral supernatant was added to cells for 24 hours. Media was refreshed without lentivirus and cells cultured for a further 24 hours before 10 µg/ml blasticidin (InvivoGen) was added to select successfully transduced cells. Expression of dCas9-KRAB was confirmed by Western blot with antibody to Cas9 (**Supplementary Fig.5**).

T-47D-dCas9-KRAB cells were seeded into 12-well plates at a density of 100,000 cells per well and incubated for 24 hours. sgRNA lentivirus at 100 µl/well was added and cells cultured

for 72 hours. Media was refreshed with 1 µg/ml puromycin (InvivoGen) to select successfully transduced cells expressing Zsgreen (visualised by fluorescent microscope). Cells were collected and RNA extracted using the RNeasy Kit (Qiagen). RNA was converted to cDNA using 2 µg with 1 µl 500 µg/ml oligo $(dT)_{15}$ primer (Promega), 1 µl 10 mM dNTP mix (Invitrogen), 4 µl 5X First Strand Buffer (Invitrogen), 1 µl 0.1 M DTT (Invitrogen), 1 µl RNase-OUT (40 U/µl) (Invitrogen) and 1 µl 200 U/µl SuperScript III Reverse Transcriptase (ThermoFisher Scientific), according to manufacturer's protocol.

qPCR

Gene expression in CRISPRi cells transduced with sgRNA lentiviral particles was measured using TaqMan assays specific to the gene of interest. A 5 µl reaction was prepared with 1:10 dilution of cDNA, 2.5 µl TaqMan Universal Master Mix II (no UNG, Applied Biosystems) and 0.25 µl 20X gene-specific TaqMan Assay (*CCDC88C* (Hs00380245_m1), *C14orf159* (Hs01061067_m1), *PPP4R3A* (Hs00215697_m1), *GAPDH* (Hs00266705_g1), all Applied Biosystems). Amplification was measured on QuantStudio v. 6 Flex Real-Time PCR System. Relative gene expression, compared to empty vector alone and normalised to *GAPDH*, was calculated using the ΔΔCT method.

Western blotting

Cells were washed in PBS and whole cell extracts were prepared by lysing cells with lysis buffer (150mM NaCl, 50mM Tris pH8.0, 1% Triton X-100, PhosStop and Complete Mini EDTA free protease inhibitors (Roche)) on ice for 5 minutes. Lysed cell suspension was collected using a cell scraper and agitated for 30 minutes at 4°C. Following centrifugation at 13,000 rpm for 25 minutes at 4°C, the whole cell lysate supernatant was aliquoted and stored at -70°C. Protein yield was quantified by Bio-Rad Protein Assay Dye Reagent Concentrate (Bio-Rad) with BSA (NEB) as standard. Whole cell lysates were subjected to SDS-PAGE on a 4-12% Bis Tris gel with MOPS buffer (Invitrogen) and transferred to a nitrocellulose membrane (0.45 µm pore size, Invitrogen). The membrane was blocked with 5% non-fat milk in TBST (1X TBS,

0.05% Tween 20) at room temperature overnight with shaking. The membrane was probed with an antibody against dCas9 ((7A9-3A3) N-Terminus-BSA Free, NBP2-36440, Novus Biologicals) diluted 1:1,000; or beta-actin (as loading control; AC-15, monoclonal, Sigma A5441) diluted 1:40,000; in 5% non-fat milk in TBST for 2 hours at room temperature. After washing 3 times with TBST, the blots were incubated with anti-mouse IgG secondary antibody (A9044, Sigma) or anti-mouse IgG (H+L) (115-035-003, Jackson Immuno Research) conjugated to horseradish peroxidase diluted 1:10,000 in TBST for 1 hour at room temperature. The blot was then washed four times with TBST and developed with ECL Detection Reagent (Bio-Rad) (**Supplementary Figure 6**).

Reporter assay

The 270 bp sequences containing rs7153397 with either the reference or alternative allele were synthesised as gBlocks by IDT (**Supplementary Data**). Reporter vectors were constructed using restriction digest cloning of gBlocks into the pGL4.23 reporter vector with minimal promoter (Promega). The plasmid and gBlocks were digested in a double restriction assay using BamHI and SalI (NEB) and dephosphorylated with Quick CIP (NEB), according to the manufacturer's protocol. Ligations were performed in a 3:1 insert:vector ratio using T4 DNA ligase (NEB), according to the manufacturer's protocol. Construct sequences were validated by Sanger sequencing (GENEWIZ, Azenta Life Sciences).

T-47D and MCF-7 cells were seeded at 16,000 cells per well of a 96-well plate. The cells were transfected once they had reached 70% confluency. Transfection was carried out using X-treme GENE HP DNA transfection reagent (Roche). Equimolar amounts (50 ng) of test pGL4 firefly vector and pRL-TK renilla luciferase control (Promega) were combined in a 3:1 reagent:DNA ratio in OptiMEM (ThermoFisher Scientific). The transfection mix was incubated at room temperature for 30 minutes and 10 µl was added per well. Untransfected, mock-transfected and pEGFP-transfected (Takara Bio Inc.) controls were included in each biological

replicate. Cells were screened for luciferase activity 48 hours after transfection using the Dual-Glo Luciferase Assay System (Promega), according to the manufacturer's protocol.

Data analysis

The Cancer Genome Atlas (TCGA) data were downloaded from the GDAC portal at: http://gdac.broadinstitute.org (release 28-January-2016) and SCAN-B from Gene Expression Omnibus (GEO), with accession ID GSE96058. The datasets were pre-processed as described previously[10,11]. Differential expression comparison of *CCDC88C* between breast cancer subtypes ER+ and ER-, and normal breast tissue for TCGA and SCAN-B, was performed using the Welch's t-test in R (v4.4.2). Survival analysis was performed using the Cox proportional hazards model in R (v4.4.2) with *P*-values estimated using the Wald-test, R package: survival (v3.8-3). Expression quantitative trait locus (eQTL) calls were compiled from the GTEx API using the get_significant_single_tissue_eqtls() function from gtexr v0.2.1.9000 in R v4.4.2. Locus plots were created in R (v4.4.1) using Gviz (v1.50.0) with custom scripts. Graphs were produced in Prism (v10.4.1). The position weighted matrix for the *FOXA1* binding site was downloaded from Rauluseviciute, et al. [12]. Correlation figures of normalized $\log_2$ DNA counts and RNA counts were replicated from MPRAflow[2]. Summary figure generation and custom post-hoc editing were performed in Adobe Illustrator v29.6.1. T-47D DNase-seq profiles were plotted from bigwig output downloaded from ENCODE 4 hg38 ENCFF085GPE. QQ plots were generated in R (v4.2.1) using the inbuilt qqnorm and qqplot R functions.

Statistical analysis

LentiMPRA

We obtained MPRAnalyze output in the format of alpha with p-values for MAD-score (a median-based variant of z-score), allele $\log_2$FC with FDR-corrected *P*-values and direction $\log_2$FC with FDR-corrected *P*-values. We used these as estimates of enhancer activity, functionality of variant and evidence of directionality of enhancer, respectively. For all measures we used an FDR corrected *P*-value with a threshold of 0.1. When FDR corrected

P-values were not produced by MPRAnalyze, these were calculated in R (v4.4.1). For the stratified analysis to estimate allelic log_2FC on forward and reverse strands separately, we plotted the quantiles of log_2FC on the forward strand against the quantiles of log_2FC on the reverse strand.

Enrichment analysis

We stratified CRS on regulatory activity (significant = alpha $P < 0.1$; non-significant = alpha $P > 0.1$) and categorised them based on absolute allele fold change. We carried out a Pearson's chi-squared test on these categories in R (v4.4.1). We carried out a two-sided Fisher's exact test in R (v4.4.1) to determine annotations with significant enrichment in differentially expressed variants, using a Bonferroni corrected *P*-value of 0.006 (8 tests).

qPCR

For the negative control sgRNAs (Scrambled_1 and Scrambled_2), we used t-tests to test H0: the relative gene expression does not differ from 1.0. To maximise the power of subsequent analyses, we then combined the negative control data and for each of the other sgRNAs we tested H0: relative gene expression does not differ from the combined negative control relative gene expression. To account for multiple testing, we used a Bonferroni corrected *P* value of 0.006 (8 tests per gene).

Reporter assay

Firefly luciferase activity was internally normalised to renilla luciferase activity, and each test condition normalised to the pGL4.23 vector with minimal promoter only. Setting the pGL4.23 vector with minimal promoter only to 1.0, for the REF and ALT putative enhancer-containing reporter gene constructs we used t-tests to test (1) H_0: the mean dual luciferase ratio does not differ from 1.0 and (2) H_0: the ALT construct does not differ from the REF construct.

REFERENCES

1. Fachal, L. *et al*. Fine-mapping of 150 breast cancer risk regions identifies 191 likely target genes. *Nat Genet* **52**, 56-73 (2020).
2. Udler, M.S., Tyrer, J. & Easton, D.F. Evaluating the power to discriminate between highly correlated SNPs in genetic association studies. *Genet Epidemiol* **34**, 463-8 (2010).
3. Gordon, M.G. *et al*. lentiMPRA and MPRAflow for high-throughput functional characterization of gene regulatory elements. *Nat Protoc* **15**, 2387-2412 (2020).
4. Eeckhoute, J. *et al*. Positive cross-regulatory loop ties GATA-3 to estrogen receptor alpha expression in breast cancer. *Cancer Res* **67**, 6477-83 (2007).
5. Hevir, N., Trost, N., Debeljak, N. & Rizner, T.L. Expression of estrogen and progesterone receptors and estrogen metabolizing enzymes in different breast cancer cell lines. *Chem Biol Interact* **191**, 206-16 (2011).
6. Ashuach, T. *et al*. MPRAnalyze: statistical framework for massively parallel reporter assays. *Genome Biol* **20**, 183 (2019).
7. Lee, D. *et al*. STARRPeaker: uniform processing and accurate identification of STARR-seq active regions. *Genome Biol* **21**, 298 (2020).
8. Inoue, F. & Ahituv, N. Decoding enhancers using massively parallel reporter assays. *Genomics* **106**, 159-164 (2015).
9. Inoue, F. *et al*. A systematic comparison reveals substantial differences in chromosomal versus episomal encoding of enhancer activity. *Genome Res* **27**, 38-52 (2017).
10. Kreimer, A., Yan, Z., Ahituv, N. & Yosef, N. Meta-analysis of massively parallel reporter assays enables prediction of regulatory function across cell types. *Hum Mutat* **40**, 1299-1313 (2019).
11. Parker, S.C. *et al*. Chromatin stretch enhancer states drive cell-specific gene regulation and harbor human disease risk variants. *Proc Natl Acad Sci U S A* **110**, 17921-6 (2013).
12. Aznar, N. *et al*. A Daple-Akt feed-forward loop enhances noncanonical Wnt signals by compartmentalizing beta-catenin. *Mol Biol Cell* **28**, 3709-3723 (2017).
13. Rosen, J.M. & Roarty, K. Paracrine signaling in mammary gland development: what can we learn about intratumoral heterogeneity? *Breast Cancer Res* **16**, 202 (2014).
14. Lindvall, C. *et al*. The Wnt signaling receptor Lrp5 is required for mammary ductal stem cell activity and Wnt1-induced tumorigenesis. *J Biol Chem* **281**, 35081-7 (2006).
15. Li, H. & Durbin, R. Fast and accurate short read alignment with Burrows-Wheeler transform. *Bioinformatics* **25**, 1754-60 (2009).
16. Frankish, A. *et al*. Gencode 2021. *Nucleic Acids Res* **49**, D916-D923 (2021).
17. Gasperini, M. *et al*. A Genome-wide Framework for Mapping Gene Regulation via Cellular Genetic Screens. *Cell* **176**, 1516 (2019).
18. Ran, F.A. *et al*. Genome engineering using the CRISPR-Cas9 system. *Nat Protoc* **8**, 2281-2308 (2013).
19. Tzelepis, K. *et al*. A CRISPR Dropout Screen Identifies Genetic Vulnerabilities and Therapeutic Targets in Acute Myeloid Leukemia. *Cell Rep* **17**, 1193-1205 (2016).

20. Xie, S., Duan, J., Li, B., Zhou, P. & Hon, G.C. Multiplexed Engineering and Analysis of Combinatorial Enhancer Activity in Single Cells. *Mol Cell* **66**, 285-299 e5 (2017).
21. Haider, S. *et al*. The transcriptomic architecture of common cancers reflects synthetic lethal interactions. *Nat Genet* **57**, 522-529 (2025).
22. Iacovacci, J. *et al*. Proteogenomic discovery of RB1-defective phenocopy in cancer predicts disease outcome, response to treatment, and therapeutic targets. *Sci Adv* **11**, eadq9495 (2025).
23. Rauluseviciute, I. *et al*. JASPAR 2024: 20th anniversary of the open-access database of transcription factor binding profiles. *Nucleic Acids Res* **52**, D174-D182 (2024).
